# A Wheel within a Wheel

**Published:** 1864-08

**Authors:** A. S. Talbert

**Affiliations:** Lexington, Ky.


### Correspondence.

### A WHEEL WITHIN A WHEEL.

LEXINGTON, KY., April 1st, 1864.

MESSRS. EDITORS:

*Gentlemen*—Supposing you to have access to the necessary opportunities for microscopic investigations, I inclose you for that purpose a deposit which I found amid the pulp of the left inferior second molar tooth. It seems to be an effort at a *tooth within a tooth!* I now could understand Ezekiel's wheels. "They turned not when they went." So of this tooth. It *went* at the same time it *stood still!*

I did not treat it with success, and finally had to extract it to learn what was the matter! The nerve was but slightly exposed by decay in the grinding surface. The patient, a young gentleman in the prime of life, some twenty-eight years of age, was very anxious the tooth should be preserved. I washed the cavity with creasote, and filled it with "Hill's Stopping," intending, if possible, thus to preserve its vitality. Next day it gave so much pain I removed the stopping, and applied the usual remedies for destroying the nerve. In a day or two my patient returned, and I removed such of the pulp as I could with an excavator. I could not find the cavities leading through the fangs. After searching in vain for them, I concluded if they were so small

I would leave that portion of the nerve in them! Again I filled with the "Stopping;" again I had to remove it. With the use of creasote and cotton for a few days, the soreness again subsided. A third time it was filled with the "Stopping." In a few hours both patient and "doctor" concluded to remove the tooth. The fangs were very much congested—the whole dentine being quite reddened. With the tooth in my hand I for the first time discovered the pulp cavity to be nearly filled with the ossific deposit which I send you, and which, though movable, could not be removed without splitting the tooth. To the under surface of it the remainder of the pulp was attached. It appears to have been developed from the centre. I having previously removed the upper covering of it, could see nothing but bone in the bottom of the cavity. During the whole of my treatment I had not perceived it to be loose in the cavity, or I should have cut the orifice more, and have taken it out, and probably have saved the tooth.

How does it look under the microscope? I remark that on account of a heavy moustache worn by the patient, and some peculiarity of the mouth, it was unusually difficult to *see* the bottom of the cavity; but my failure was a mystery to me, solved only by the extraction of the tooth.

I am, &c., A. S. TALBERT.

